# Pregnancy in Obese Mice Protects Selectively against Visceral Adiposity and Is Associated with Increased Adipocyte Estrogen Signalling

**DOI:** 10.1371/journal.pone.0094680

**Published:** 2014-04-14

**Authors:** Silvia M. A. Pedroni, Sophie Turban, Tiina Kipari, Donald R. Dunbar, Kerry McInnes, Philippa T. K. Saunders, Nicholas M. Morton, Jane E. Norman

**Affiliations:** 1 Tommy's Centre for Maternal and Fetal Health, The University of Edinburgh, Queen's Medical Research Institute, Edinburgh, United Kingdom; 2 MRC Centre for Reproductive Health, The University of Edinburgh, Queen's Medical Research Institute, Edinburgh, United Kingdom; 3 Endocrinology Unit, University/BHF Centre for Cardiovascular Science, The University of Edinburgh, Queen's Medical Research Institute, Edinburgh, United Kingdom; 4 Molecular Metabolism Group, University/BHF Centre for Cardiovascular Science, The University of Edinburgh, Queen's Medical Research Institute, Edinburgh, United Kingdom; State University of Rio de Janeiro, Biomedical Center, Institute of Biology, Brazil

## Abstract

Maternal obesity is linked with increased adverse pregnancy outcomes for both mother and child. The metabolic impact of excessive fat within the context of pregnancy is not fully understood. We used a mouse model of high fat (HF) feeding to induce maternal obesity to identify adipose tissue-mediated mechanisms driving metabolic dysfunction in pregnant and non-pregnant obese mice. As expected, chronic HF-feeding for 12 weeks preceding pregnancy increased peripheral (subcutaneous) and visceral (mesenteric) fat mass. However, unexpectedly at late gestation (E18.5) HF-fed mice exhibited a remarkable normalization of visceral but not peripheral adiposity, with a 53% reduction in non-pregnant visceral fat mass expressed as a proportion of body weight (P<0.001). In contrast, in control animals, pregnancy had no effect on visceral fat mass proportion. Obesity exaggerated glucose intolerance at mid-pregnancy (E14.5). However by E18.5, there were no differences, in glucose tolerance between obese and control mice. Transcriptomic analysis of visceral fat from HF-fed dams at E18.5 revealed reduced expression of genes involved in *de novo* lipogenesis (diacylglycerol O-acyltransferase 2 - Dgat2) and inflammation (chemokine C-C motif ligand 2 - Ccl2) and upregulation of estrogen receptor α (*ERα*) compared to HF non pregnant. Attenuation of adipose inflammation was functionally confirmed by a 45% reduction of CD11b^+^CD11c^+^ adipose tissue macrophages (expressed as a proportion of all stromal vascular fraction cells) in HF pregnant compared to HF non pregnant animals (P<0.001). An ERα selective agonist suppressed both *de novo* lipogenesis and expression of lipogenic genes in adipocytes *in vitro*. These data show that, in a HF model of maternal obesity, late gestation is associated with amelioration of visceral fat hypertrophy, inflammation and glucose intolerance, and suggest that these effects are mediated in part by elevated visceral adipocyte ERα signaling.

## Introduction

Obesity is an important risk factor for adverse pregnancy outcomes for the mother, (eg preeclampsia, gestational diabetes, caesarean section) and the baby (eg miscarriage, stillbirth and macrosomia) [1]. Pregnancy itself alters maternal metabolism to accomodate the increasing metabolic need of the fetus. During the first trimester, pregnancy is characterized by increased deposition of fat and normal or improved glucose/insulin homeostasis, followed by increased adipose tissue inflammation, adipocytokine production and adipose tissue insulin resistance in the third trimester [2]–[9]. Adipose tissue insulin resistance may, via elevation of plasma fatty acids, further contribute to the observed increase in hepatic glucose production (HGP) and hepatic insulin resistance [4], [10], [11]. Given the known association between hyperglycaemia, hyperinsulinaemia and adverse pregnancy outcome shown in the Hyperglycemia and Adverse Pregnancy Outcomes (HAPO) study and others [12], [13], it is likely that metabolic dysfunction is causal in the link between maternal obesity and pregnancy complications. Although many effect of pregnancy on maternal metabolism have been defined, the interactions between obesity and pregnancy on adipose tissue function, which plays an essential role on metabolism, are incompletely understood. Improvements in understanding the impact of pregnancy on adipose tissue metabolism in obese pregnant women could aid the development of therapies to reduce the risk of adverse pregnancy outcome in obesity. In order to address this issue, we performed a detailed phenotyping study using a high fat (HF) fed murine model of obesity, together with a transcriptomic and an *in vitro* functional analysis of visceral fat to identify adipose tissue-mediated mechanisms driving metabolic dysfunction in obese and lean pregnancy.

## Methods

### Animals

All experiments were approved by The University of Edinburgh ethics committee and conducted according to the U.K. Animals (Scientific Procedures) Act 1986. Five week old female mice (C57Bl/6J) obtained from different litters and with different mothers, were fed with either a high fat diet (Research Diets D12331: 58%kcal fat, 25.5%kcal sucrose and 16.4%kcal protein) (HF) or a low fat control diet (Research Diets D12328: 10.5%kcal fat, 73%kcal corn starch and 16.4%kcal protein) (control) for 12 weeks before being mated with males who had been fed with chow diet. The day on which a plug was detected was considered day 0.5 of pregnancy. Pregnant females were weighed on gestational days 10.5, 14.5 and 18.5 (E10.5, 14.5 and 18.5), non pregnant mice were also weighed. Mice were sedated prior to sacrifice in the morning, and blood was collected by aortic puncture, in sedated mice, with a 28 G needle into an EDTA-coated 1 ml tube. Subcutaneous (around the thigh) and visceral (gonadal and mesenteric) fat were recovered and weighed, visible lymph nodes were dissected from fat tissue and samples snap frozen in liquid nitrogen. In a parallel group of mice, a glucose tolerance test was performed at E14.5 and E18.5 on fasted mice (see below).

### Oral glucose tolerance test (GTT)

After fasting for 6 , 2 mg/g body weight D-glucose (25% v/w) was administered by oral gavage. Blood samples were collected by tail bleeding at 0, 15, 30, 60 and 120-minute intervals after the administration of glucose.

### ELISA

Plasma leptin (90030-Crystal chem, Downers Grove, USA; assay range 0.2–12.8 ng/mL), high molecular weight adiponectin (47-ADPMS-E01-Alpco, Salem, USA; assay range 0.125–8.0 ng/mL), estradiol (ES180S-100-CalBiotech, Spring Valley, USA; assay range 3–300 pg/mL) [14] and insulin (90080-Crystal chem, Downers Grove, USA; Wide Range Assay: 0.1–12.8 ng/mL) were measured by ELISA using the kits denoted here.

### Microarray analysis

Visceral adipose tissue RNA was prepared using Qiagen RNeasy Mini kits (QIAGEN, Crawley-West Sussex, U.K.). RNA integrity was calculated using a RNA 6000 Nano chip on an Agilent 2100 bioanalyzer (Agilent Technologies, Palo Alto, CA). Microarray analysis was performed at the ARK Genomics Facility (Roslin Institute, Edinburgh, U.K.) on samples with a RIN of >8.5. Array analysis was performed using standard protocols. Briefly, samples were hybridized to the Affymetrix Mouse Genome 430-2.0 GeneChip which recognises 39,000 transcripts. Array data were extracted through the GeneChip Operating Software (GCOS), and CEL files were imported into Bioconductor and normalized by robust multi-array average in the “Affy” module. We used the Limma and Rank products (RankProd) packages to perform statistical analysis. Spotfire DecisionSite (http://spotfire.tibco.com/) was used to plot gene expression data. We performed pathway analysis using DAVID (http://david.abcc.ncifcrf.gov/), Webgestalt (http://bioinfo.vanderbilt.edu/webgestalt/) and Metacore (from GeneGo Inc., St. Joseph, MI, USA) tools for genes with Rank Product P-value of <0.05, expression level >100 and fold change ±1.5. Microarray data were deposited in the Gene Expression Omnibus (GEO) with accession number GSE48811.

### Quantitative RT-PCR

600 ng of total RNA, from visceral adipose tissue, was reverse transcribed using the cDNA Synthesis Kit (4368813-Applied Biosystems, Carlsbad, US). We analysed levels of gene-specific mRNA using an ABI 7900HT (Applied Biosystems, Hill, U.K.) with inventoried probes and primer sets included in Table 1 (Applied Biosystems, Hill, U.K.) and expressed values normalized against cyclophilin A mRNA levels.

**Table 1:** List of Taqman primer and probes used for the study.

Gene	Assay ID
Mouse Cyclophilin A	Mm02342429_g1
Mouse FASN	Mm00662319_m1
Mouse Rbp4	Mm00803266_m1
Mouse ERα	Mm00433149_m1
Mouse SCD1	Mm00772290_m1
Mouse ME1	Mm00782380_s1
Mouse Dgat2	Mm01273905_m1
Mouse Hsd17b12	Mm00479916_m1
Mouse TNFα	Mm00443258_m1
Mouse MCP1	Mm00441242_m1
Mouse Rdh11	Mm00458129_m1

### Western blotting

Mesenteric adipose tissue was homogenized in ice-cold lysis buffer (50 mmol/L Tris, pH 7.4, 0.27 mol/L sucrose, 1 mmol/L sodium orthovanadate, pH 10, 1 mmol/L EDTA, 1 mmol/L EGTA, 10 mmol/L sodium β-glycerophosphate, 50 mmol/L NaF, 5 mmol/L sodium pyrophosphate, 1% [w/v] Triton X-100, 0.1% [v/v] 2-mercaptoethanol and one tablet of complete TM protease inhibitor (Roche, Burgess Hill, U.K.). Thereafter, 50 µg of protein was run on 4–12% Bis-Tris gels for Western blotting. To measure plasma concentrations of Rbp4 we denatured 1 µl of plasma in standard loading buffer. Protein signals were visualized using enhanced chemiluminescence (Pierce Biotechnology, Rockford, IL) by exposure to Amersham HyperfilmTH ECL film (Amersham). We used primary antibodies raised against ERα (8644S Cell Signaling Technologies, U.K), β-tubulin (2128S Cell Signaling Technologies, U.K.) and Rbp4 (A0040-Dako, U.K.) and visualized the bands by adding horseradish peroxidase anti-rabbit secondary antibody (Cell Signaling Technology).

### Adipose tissue fractionation

Gonadal fat was digested in Krebs–Ringer solution with 2 mg/mL collagenase type I (Worthington Biochemicals, NJ, USA) at 37°C for 1 h, filtered through 250-micron size exclusion mesh and centrifuged at 600xg for 10 minutes to separate adipocytes from stromal vascular cells (SVCs). Erythrocytes were lysed by re-suspending the SVCs in 1 mL of erythrocyte lysis buffer (Sigma Aldrich, Dorset, U.K.) for 5 min. Cells were then collected by centrifugation and re-suspended in flow cytometry buffer.

### Flow cytometry of pro-inflammatory macrophages in adipose tissue

For flow cytometry, 5×10^5^ of gonadal fat stromal vascular cells were pre-incubated in 100 µL PBS with 1 µg/mL FcR block (BD Biosciences, Oxford, U.K.) and then incubated with 0.2 µg each of rat anti-mouse– CD11b FITC, and hamster anti-mouse-CD11c PE (Caltag, Invitrogen, Paisley, U.K.) in PBS with 10% mouse serum (Sigma Aldrich, Dorset, U.K.) for 30 min at 4°C in the dark. Cells were sorted using a FACScalibur (BD Biosciences) flow cytometer and analyzed using FlowJo 8.0 software (Treestar Inc., Ashland, OR).

### Female clonal adipocyte Chub-S7 cell line

An aliquot of immortalized primary human preadipocytes (subsequently termed the Chub-S7 cell line), a gift from Dr Christian Darimont (Nestlé Research Centre, Lausanne, Switzerland) was differentiated according to published protocols [15]. After 17 days of differentiation, fully differentiated Chub-S7 cells were treated with the estrogen receptor alpha agonist 4,4′,4″-(4-Propyl-[1H]-pyrazole-1,3,5-triyl) trisphenol (PPT) (1426-Tocris, Bristol, U.K.) in DMEM media (D6046-Sigma-Aldrich). A vehicle DMSO control was included. Samples were incubated for 6 hours in a cell culture incubator at 37°C with 5%CO_2_ in 6 wells plates. Messenger RNA levels were measured in extracted total RNA as described above.

### Measurement of *de novo* lipogenesis in isolated primary murine adipocytes

We quantified lipogenesis in isolated fat cells as previously described [16]. Briefly, isolated adipocytes from gonadal adipose tissue from groups of four non pregnant control female mice were pooled and incubated for 12 hours with vehicle or 10 nM PPT in a Krebs phosphate buffer. Following the incubation of adipocytes, the cells were washed twice with 10 volumes of Krebs phosphate prior to starting incubation with ^3^H Glucose to a final concentration of 0.5 µCi/ml with or without the presence of 10 nM insulin. Typical Media/Cells ratio was 5/1 by volume. Cells were lysed by adding.

1 µl/10 ml experimental medium of 6 M H_2_SO_4_ and vortexing. Organic scintillant (POPOP+PPO in toluene; Fluka 327123) was added (2 volumes/1 volumes of media), without mixing, and then left for at least 2 hours without agitation to allow the lipids to diffuse into the organic layer before counts were measured in a scintillation counter (Tri-Carb 2100 TR Liquid Scinillation Analyser, Packard). Lipid were extracted using a solution composed by 4v Isopropanol, 1v Heptane and 1v Sulfuric acid 1N. We calculated relative lipogenesis by determining radioactive glucose incorporation into total lipid content.

### Statistical analysis

Data are presented as the mean ± standard error of the mean (S.E.M). Statistical analysis was performed using SigmaStat 3.5 and graphs were created using Graph Pad (GraphPad Prism 4). Unless otherwise stated two-way ANOVA (Holm-Sidak, pairwise multiple comparison) was performed with pregnancy and diet status (HF or control) as independent variables. A P value of <0.05 was considered to show a statistically significant difference between the groups.

## Results

### Normalization of visceral fat mass at late gestation in HF mice

**Figure 1:**
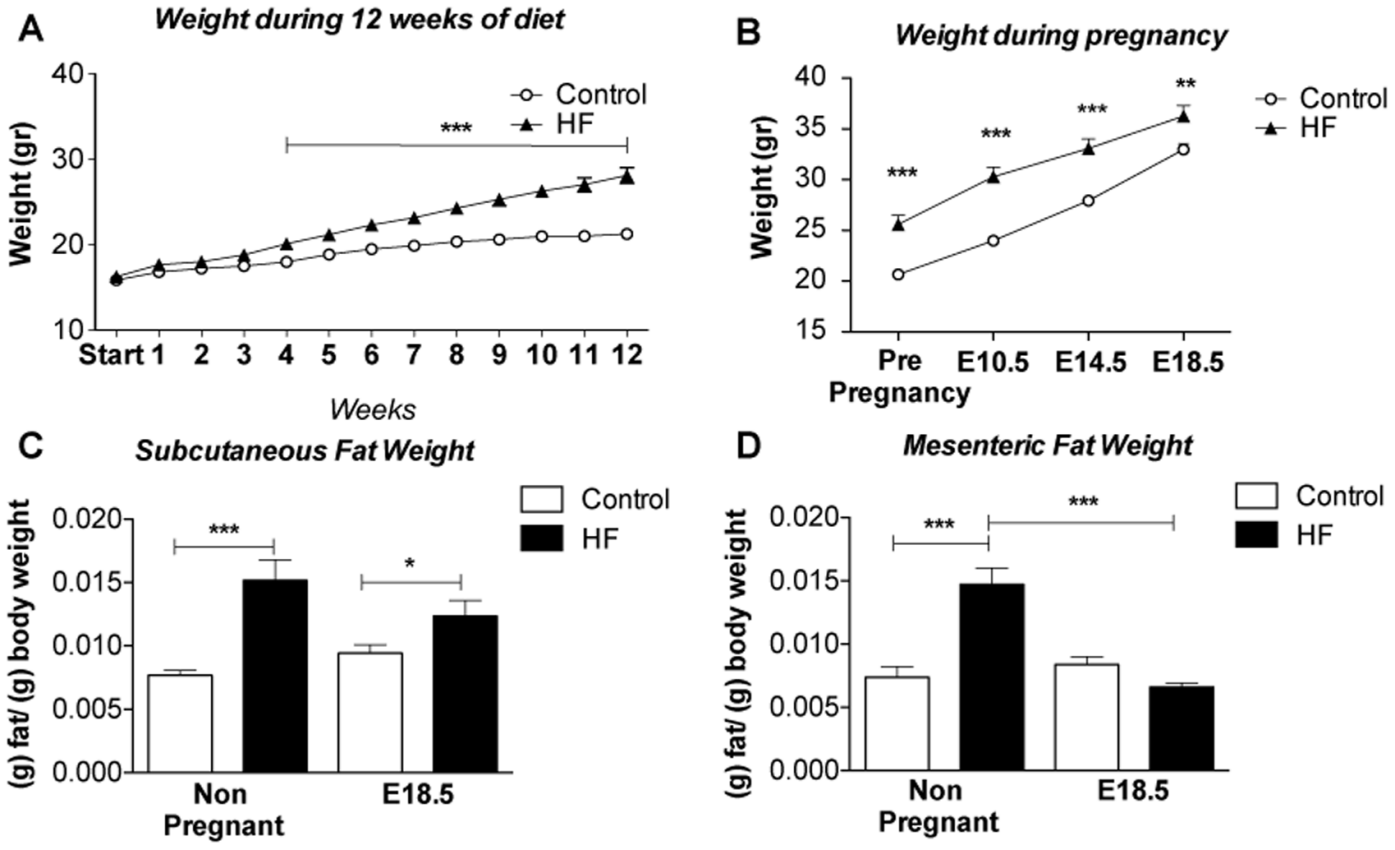
Pregnancy results in increased weight gain and selective changes in fat depots in HF-fed mice. **A**. Weight during 12 weeks treatment of HF diet in HF mice and control diet in control animals (n = 19–30); **B**. Weight during pregnancy (n = 8–16), note that both control and HF-fed (obese) mice gained weight, that differences between the two groups were maintained throughout pregnancy but that differences between them were smaller by E18.5; **C**. subcutaneous fat weight in g/g of body fat (n = 8–16); **D**. mesenteric fat weight in g/g of body fat (n = 8–16), note there is no difference in the weight of this depot between control and HF-fed individuals at E18.5; Statistical analysis performed using two way ANOVA * = P≤0.05, ** = P≤0.01, *** = P≤0.001 comparing HF and relevant control.

High fat (HF) feeding for 12 weeks resulted in a 30% increase in body weight in female mice (Figure 1A) which was in agreement with previous studies from our laboratory using this model [17]. Although at time of conception mice on the control diet weighed less than their HF counterparts they gained more weight (as a proportion of body fat) than HF in pregnancy (60% vs 42% from conception to E18.5) and there was a convergence in weight by the end of pregnancy (Figure 1B). Despite this convergence, the HF remained heavier than controls at E18.5. In both pregnant and non-pregnant groups, HF-feeding resulted in a significant increase in subcutaneous fat mass (as a fraction of total body weight) that was largely maintained throughout pregnancy (Figure 1C). In contrast, there was a significant and selective net reduction (a −53% reduction) in mesenteric fat (as a fraction of total body weight) mass in HF-fed pregnant mice leading to no observable differences in mesenteric fat mass between HF and control mice by E18.5 (Figure 1D).

### HF pregnant mice exhibit a normalised adipokine profile at late-stage pregnancy

To determine whether the reduction in visceral fat mass found in HF pregnant mice was associated with an improvement in adipokine secretion and metabolic profile, we measured plasma levels of leptin and adiponectin^HMW^ (Table 2). As expected, prior to pregnancy, HF mice had greater plasma leptin and lower adiponectin^HMW^ levels than control mice (Table 2). During pregnancy, circulating leptin increased in both lean and HF pregnant animals, perhaps reflecting leptin production from other organs, such as placenta [18] (Table 2). Notably, during pregnancy, plasma adiponectin decreased in control diet-fed pregnant mice, but not in HF mice compared to non pregnant animals so that by the end of pregnancy there were no differences in adiponectin^HMW^ levels between the HF and control groups (Table 2).

**Table 2:** Effect of HF diet and pregnancy on concentrations of adipokines in the circulation.

Protein	Control Non Pregnant	HF Non Pregnant	Control Pregnant	HF Pregnant
**Leptin (ng/mL)**	14.5±2.29	68.4±6.72**	120.2±8.94^†††^	136.3±26.37^§§^
**Adiponectin^HMW^ (µg/mL)**	12.4±0.91	9.8±0.33***	6.9±0.53^†††^	6.39±0.42

Leptin (n = 8–15) and Adiponectin^HMW^ (n = 8 in each group). Statistical analysis performed using two way ANOVA, comparisons between HF non pregnant and control non pregnant: ** = P<0.01, *** = P<0.001, comparisons between control pregnant and control non pregnant: ††† = P<0.001, comparisons between HF pregnant and HF non pregnant: §§ = P<0.01.

### Metabolic dysfunction converges with that of lean pregnant mice in late gestation HF mice

**Figure 2:**
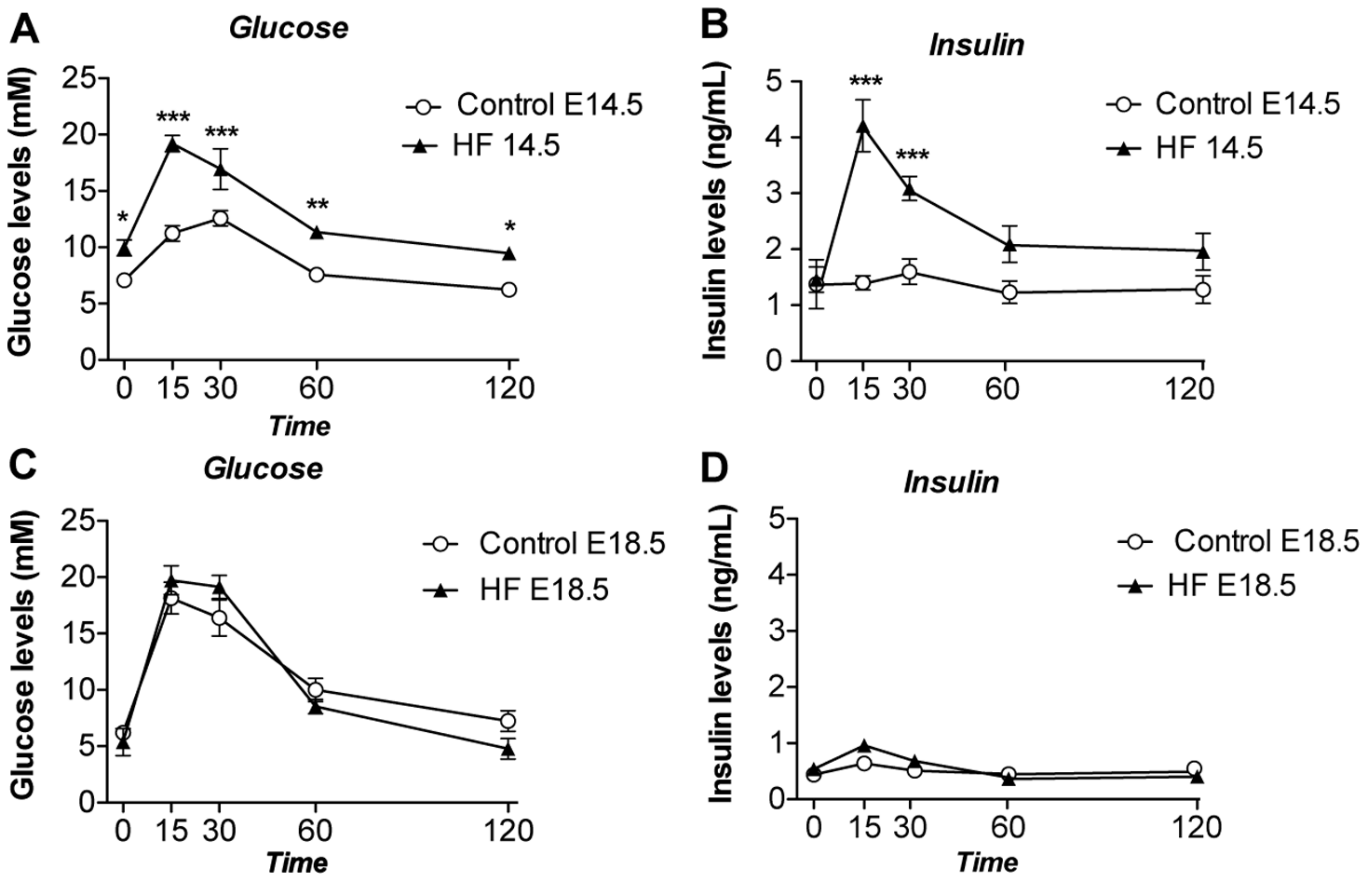
Effect of HF diet and pregnancy on glucose and insulin in response to a 2 mg/g body weight D-glucose tolerance test. A. Glucose plasma levels at E14.5 (n = 5–12), B. Insulin plasma levels at E14.5 (n = 5–12), C. Glucose plasma levels at E18.5 (n = 6–7), D. Insulin plasma levels at E18.5 (n = 6–7). Statistical analysis comparing HF with a similar time point in the control group performed using two way ANOVA * = P<0.05, ** = P≤0.01, *** = P≤0.001.

HF-fed pregnant mice displayed pronounced glucose intolerance and apparent insulin resistance compared with control diet-fed pregnant mice at E14.5 (Figure 2A and 2B). However, at E18.5, control diet-fed mice showed a significant worsening of glucose tolerance whereas HF-fed mice showed no further deterioration in glucose tolerance compared to E14.5, leading to an unexpected convergence in the glucose homeostasis profile of the two groups (Figure 2C and 2D). Remarkably, the convergence in glucose tolerance was accompanied by a correction of the hyperinsulinaemia exhibited by the HF pregnant mice at mid gestation (compare Figure 2B and 2D) suggesting if anything an enhancement in whole body insulin sensitivity in the HF-fed E18.5 compared to the HF-fed E14.5 stages. There were no differences in fasting glucose/insulin ratio comparing control and HF-fed pregnant mice either at E14.5 (ratios of 7.75±1.1 and 7.5±1.2 respectively) or at E18.5 (ratios of 15.1±5.1 and 15.9±2.7 respectively).

### Transcriptomic analysis reveals gene pathways linked to reduced visceral adiposity and inflammation in HF pregnant mice

**Figure 3:**
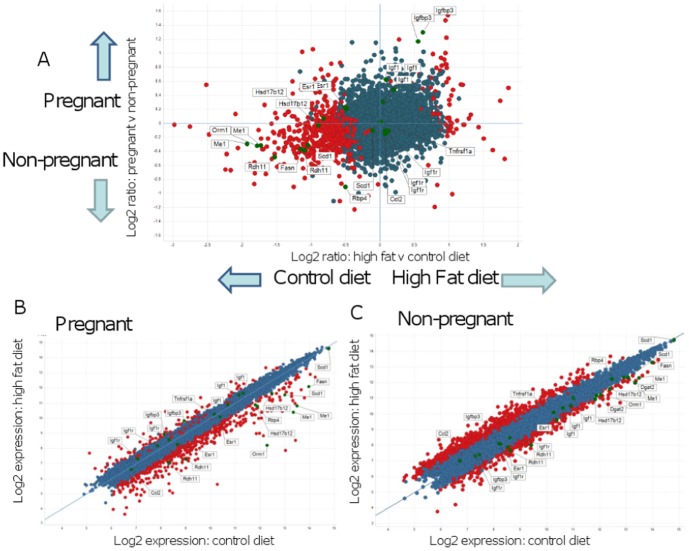
Array analysis of mesenteric fat depots from control and HF-fed obese mice using Spotfire Decision Site. **A**. Log (base 2) ratios of gene expression intensities in pregnant and non-pregnant mice on the high fat and control diets. The y-axis shows the comparison of data from pregnant and non-pregnant mice regardless of diet. The x-axis compares high fat and control diets regardless of pregnancy status. Red spots represent genes that are significantly differentially expressed in pregnant mice on HF compared to control diet. Genes expressed below the arbitrary threshold (100) throughout the experiment were removed for clarity. Several genes of interest with higher expression in pregnant mice and on a high fat diet are marked and labelled. B. and C. Log (base 2) gene expression intensities in (**B**) Pregnant mice and (**C**) Non-pregnant mice on the high fat and control diets. For each group (pregnant or non pregnant), the y-axis shows the log2 expression in HF mice and the x-axis shows log2 expression in control mice. Red spots represent genes that are significantly differentially expressed in high fat diet compared to control diet for each pregnancy status. Several genes of interest discussed in the text are marked and labelled on graphs. Genes expressed below the arbitrary threshold (100) throughout the experiment were removed for clarity.

Microarray analysis was performed to understand the molecular mechanisms associated with altered visceral fat mass in HF pregnant mice. We used Spotfire DecisionSite to show the distribution of the differentially expressed genes in our control and HF, both non pregnant and E18.5 (Figure 3). We aimed to identify specific genes altered by the interaction between diet and pregnancy.

Particular genes of interest are marked and labelled on Figure 3 and summarized in Table 3.

**Table 3:** Pathway analysis of differentially expressed genes in visceral adipose tissue of HF-fed pregnant mice.

**Up-regulated genes**			
Pathway	Gene symbol	Gene name	Mean fold change
Secreated Protein	*Igf1*	Insulin growth factor 1	1.6
	*Igfbp3*	Insulin growth factor binding protein 3	3.1
Estrogenic Signalling	*Esr1 (ERα)*	Estrogen receptorα	1.4
	*HSD17b12*	Hydroxysteroid (17-beta) dehydrogenase 12	1.5
**Down-regulated genes**			
Pathway	Gene symbol	Gene name	Mean fold change
De novo lipogenesis and lipid storage	*ME1*	Malic enzyme 1	−2.9
	*FASN*	Fatty acid synthase	−2.3
	*SCD1*	Stearoyl-CoA denaturase 1	−1.4
	*Dgat2*	Diacylglycerol O-acyltransferase2	−2.2
Inflammation	*Mcp1*	Monocyte chemoattactant protein 1	−1.3
	*TNFα*	Tumor necrosis factor α	−1.1
	*Orm1*	Orosomucoid 1	−8
Retinol metabolism	*Rbp4*	Retinol binding protein 4	−2.7
	*Rdh11*	Retinol dehydrogenase 11	−1.6

Highly up or down-regulated pathways are listed along with individual genes. Fold changes between these two groups are shown for clarity. Pathways/fold changes were analysed with David, Websgestalt and Metacore.

Bioinformatics pathway analysis (Table 3) suggested that expression of genes involved in *de novo* lipogenesis and lipid storage (*Me1, Fasn, Scd1* and *Dgat2*), inflammation (*Ccl2, Tnfα*) and retinol metabolism (*Rbp4, Rdh11*) were down-regulated in visceral adipose tissue of HF-fed pregnant, compared with HF non pregnant mice. In contrast, there was an increased expression of genes involved in estrogen biosynthesis/action (*Esr1, Hsd17β12*) (Table 3).

**Figure 4:**
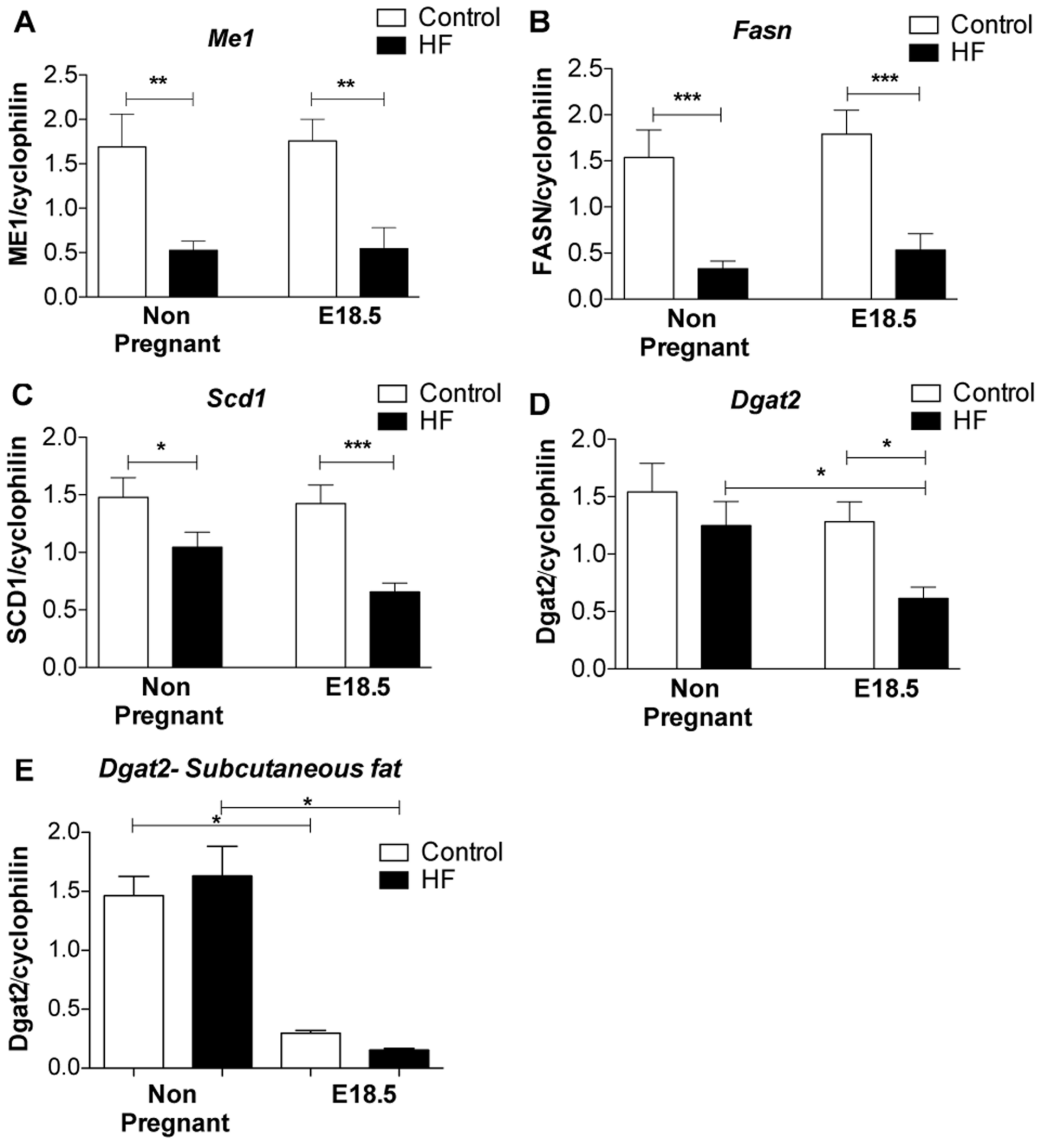
Expression of mRNAs encoded by genes involved in *de novo* lipogenesis. **A**. *Me1*, **B**. *Fasn*, **C**. *Scd1* and **D**. *Dgat2* mRNA levels in mesenteric fat; **E**. *Dgat2* gene expression levels in subcutaneous fat. Statistical analysis using two way ANOVA * = P<0.05, **P≤0.01, ***P≤0.001 (n = 8 in each group).

We performed qRT-PCR to validate the array findings. We found that genes involved in *de novo* lipogenesis (*Me1*, *Fasn* and *Scd1*) were down regulated by HF feeding independently of pregnancy in visceral fat (Figure 4A–C). However *Dgat2*, which plays a key role in triglyceride storage, was selectively decreased in visceral fat of HF-fed pregnant mice compared to both non-pregnant HF-fed animals and control diet-fed pregnant mice (Figure 4.D). This effect was in addition to the effects of pregnancy itself in decreasing Dgat2 mRNA levels in both subcutaneous and mesenteric fat (Figure 4E).

**Figure 5:**
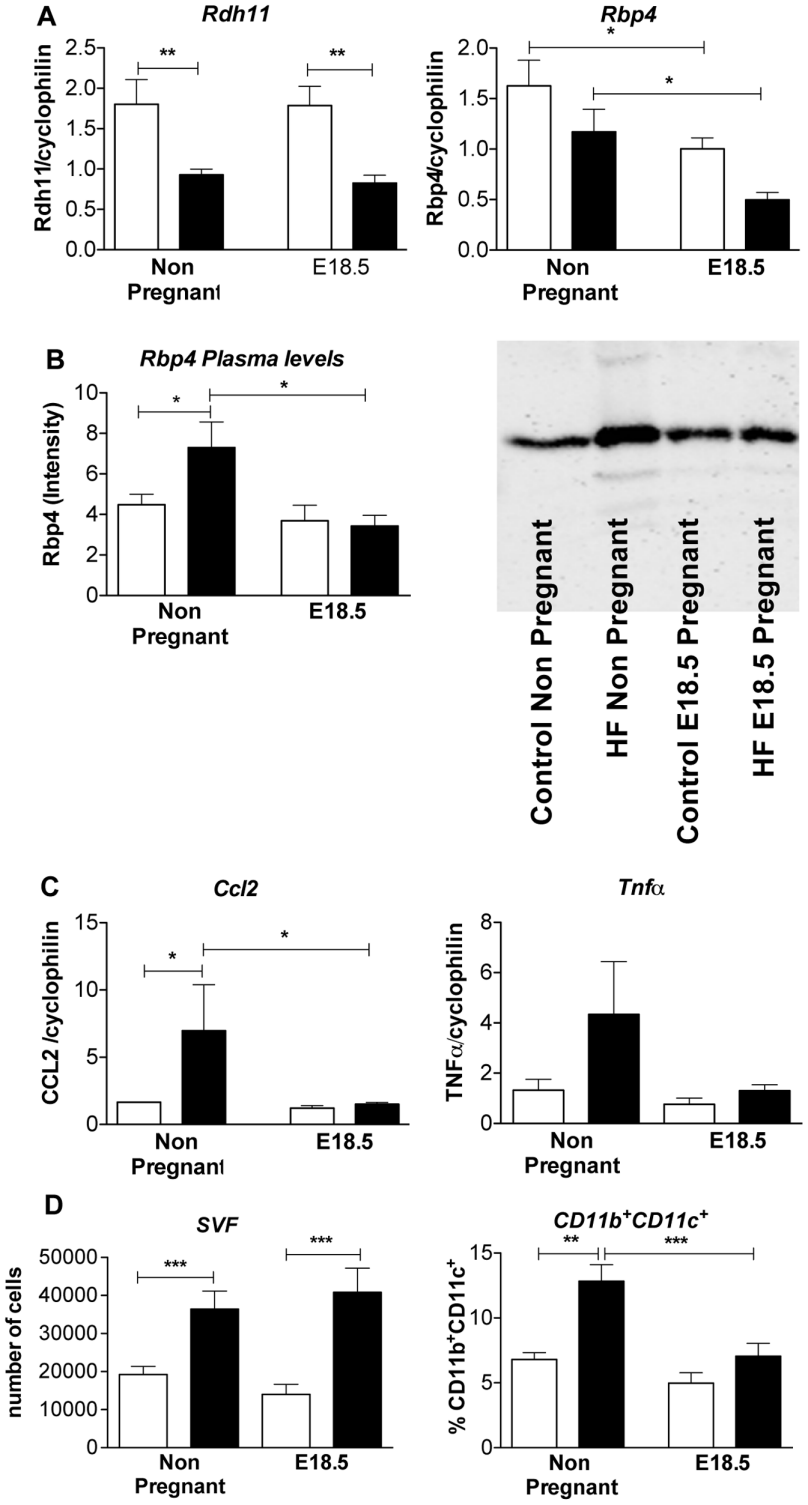
Changes in expression of genes involved in retinol metabolism and inflammation. **A**. expression of genes involved in retinol metabolism (*Rdh11* and *Rbp4* mRNA); **B**. Rbp4 circulating levels determined by Western blot in plasma; **C**. genes involved in inflammation (*Mcp1* and *TNFα* mRNA); **D**. Number of cells in stromal vascular fraction (SVF) and quantity of pro-inflammatory CD11b^+^ and CD11c^+^ double positive cells in gonadal fat determined by FACS analysis. Statistical analysis using two way ANOVA * = P<0.05, **P≤0.01, ***P≤0.001 (n = 8 in each group).

*Rdh11* mRNA was down regulated in HF mice independently of pregnancy (Figure 5A). In contrast, mesenteric fat *Rbp4* mRNA level was down regulated by pregnancy but not HF feeding (Figure 5A). A decline in Rbp4 plasma levels in pregnancy was limited to HF mice, with increased plasma Rbp4 levels in HF non-pregnant mice compared to non pregnant controls (Figure 5B).

*Ccl2* mRNA was up regulated in mesenteric fat of HF compared with control non pregnant mice (Figure 5A). A similar trend was observed with *Tnfα*. Pregnancy suppressed *Ccl2* mRNA levels in visceral fat of HF pregnant mice, leading to convergence of *Ccl2* and *Tnfα* mRNA expression in HF and control animals by E18.5 (Figure 5C). Consistent with reduced adipose inflammatory burden, pro-inflammatory CD11b^+^/CD11c^+^ macrophage density in gonadal fat was increased with HF in non-pregnant mice but converged with, and was comparable to, density in control diet-fed pregnant mice by E18.5 (Figure 5D).

### Reduced visceral adiposity in HF pregnancy is associated with increased adipocyte estrogens signalling

**Figure 6:**
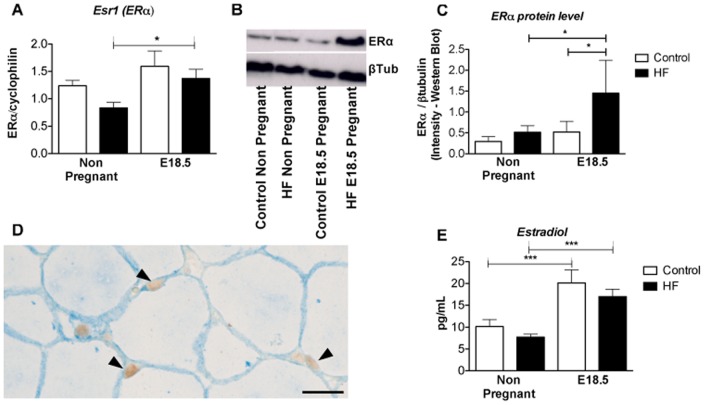
Effect of HF diet and pregnancy on visceral adipose tissue estrogen receptor expression and circulating estrogen levels. A. *Esr1* (ERα) mRNA; B, C. Western analysis of ERα in visceral adipose tissue; D. Immunolocalisation of ERα to adipocyte nuclei (arrowheads) in mesenteric/visceral adipose tissue; E. Concentrations of estradiol in plasma. Statistical analysis calculated using two way ANOVA * = P<0.05, **P≤0.01, ***P≤0.001 (n = 8 in each group).

qRT-PCR analysis revealed that ERα mRNA levels were was higher in visceral fat of HF-fed E18.5 compared with non-pregnant HF-fed mice (Figure 6A). These results were mirrored by the pattern of expression of ERα protein (Figure 6B and C). Immunohistochemistry localised the expression of ERα to adipocyte nuclei (Figure 6D). Notably, pregnancy was associated with increased plasma estrogen levels, as shown by others [19]. HF feeding alone had no impact on estradiol levels (Figure 6E).

### ERα activation suppresses lipogenesis in murine primary and human clonal adipocytes

**Figure 7:**
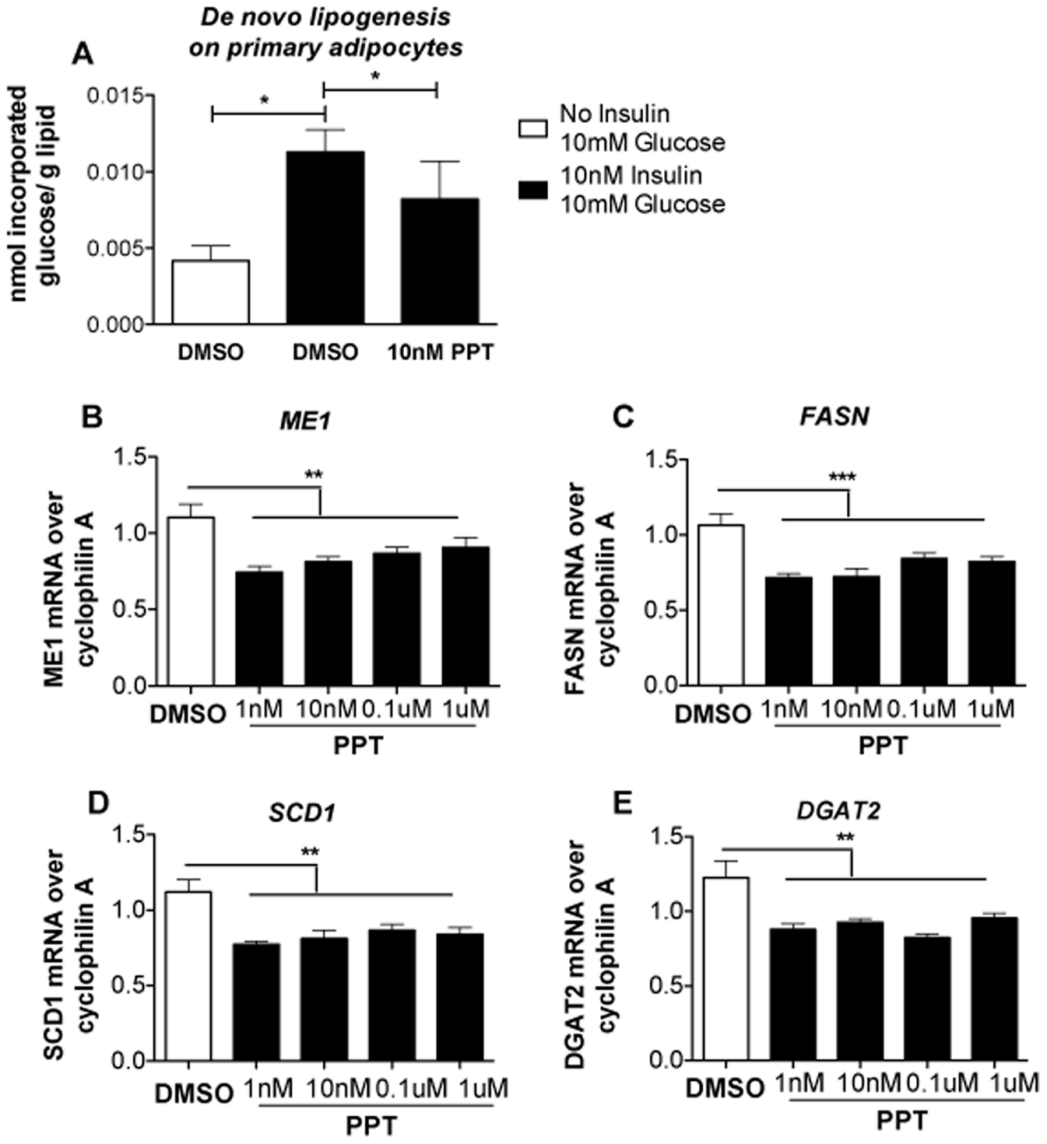
Effect of the ERα-selective agonist PPT on *de novo* lipogenesis of primary adipocytes and expression levels of relevant genes in fully differentiated Chub-S7. A. effect of ERα activation mediated by treatment of primary adipocytes with 10 nM PPT, on de novo lipogenesis in presence of 10 nM insulin. Effect of different concentration of PPT treatment (1 nM, 10 nM, 0.1 µM and 1 µM) on gene expression of B. *Me1*, C. *FASN*, D. *SCD1* and E. *Dgat2* (n = 6 in each groups). Normality of data distribution was confirmed using the Kolmogorv-Smirnov test. Statistical analysis was performed using the Kruskal-Wallis Test with Dunns as a post hoc test: comparison of pairs of columns* = P<0.05, ** = P<0.01 and *** = P<0.001.

Increased estrogen signalling is linked to lower visceral adiposity in females [20]–[22] and suppresses inflammation [23]. We reasoned that the increased estrogenic signalling found in late gestation in the HF-fed group might drive the observed transcriptome changes and selectively reduce visceral fat mass. To test this hypothesis we treated primary female mouse adipocytes with the ERα selective agonist PPT. In the presence of 10 nM insulin, 10 nM PPT treatment decreased *de novo* lipogenesis (Figure 7A). Moreover, in fully differentiated female human Chub-S7 adipocyte cells, PPT suppressed key genes for *de novo* lipid synthesis *Me1*, *Fasn*, *Scd1* and *Dgat 2* (Figure 7B–E).

## Discussion

Our key and unexpected observation is the inhibition of the progressive worsening of visceral adiposity, inflammation and metabolic dysfunction observed during normal pregnancy when mice are fed a HF diet prior to and throughout gestation. The protection and reversal of visceral adiposity happens at a late stage in pregnancy and occurs despite an exaggerated deterioration in metabolic function at mid-gestation in HF-fed mice. We suggest that reduced visceral adiposity might be mediated, in part, through direct actions of estrogen to suppress *de novo* lipogenesis.

Our results showed that prior to pregnancy, HF animals display greater mesenteric fat weight and glucose intolerance than control mice. At E14.5 (mid gestation), impaired glucose tolerance in HF compared with control animals is maintained. However, by late gestation (E18.5), glucose tolerance has not worsened further in the HF-fed group and indeed insulin levels are reduced, suggesting an increase in whole body insulin sensitivity or an improvement in pancreatic beta cell function. Further, visceral fat mass, pro-inflammatory cytokine gene expression and proinflammatory adipose tissue macrophage density are reduced in HF-fed mice at E18.5. The pregnancy related reduction in visceral fat mass observed in parallel with maintained subcutaneous fat expansion in HF mice contrasts with the observed increase in adipose tissue expansion in pregnant rats treated with a HF diet [29], [30] and with the increased general adiposity observed in a mouse model treated with a more moderate high fat diet than the one used in the study reported here [24]. Although our HF mice continued to gain weight in pregnancy, and weighed more than the control mice at term, their pregnancy weight gain as a proportion of body weight was lower than that for control mice in pregnancy. We believe that the convergence in body weight observed in HF-fed and control mice cannot be due to differences in fetal weight, given that King et al (using the mouse model presented here) showed minimal differences in birth weight in female offspring of HF fed mice (limited to 6% in female fetuses, with no effect observed in the birth weight of male fetuses) [17]. Furthermore, we believe that the convergence in maternal weight by E18.5 is not due to calorie restriction in HF mice because previously published work by King et al has shown that energy intake is similar in control and HF pregnant mice [17].

Increased estrogen signalling through ERα drives an improved metabolic phenotype and a favourable fat distribution in rodents and humans in males and non-pregnant females [21], [22]. Our results reveal a novel role for a selective increase in estrogen signalling through increased ERα expression in visceral adipocytes of pregnant animals given HF diet. These changes in ERα occur during a period of pregnancy characterised by increased circulating concentrations of estradiol. Our transcriptomic and functional studies in Chub-S7 cells and primary adipocytes, in which PPT treatment (acting through ERα) inhibits *de novo* lipogenesis, are supported by similar finding in PPT-treated 3T3-L1 adipocytes [25]. Our data are also consistent with the effect of estradiol treatment to decrease lipogenesis and triglyceride storage [26], and with the phenotype of increased lipogenesis in ERα^−/−^ mice [27]–[28]. Thus we hypothesize that estrogen plays a fundamental role in suppression of lipogenic pathways in visceral adipose tissue of HF pregnant mice. The driver to increased ERα expression is unclear, but a likely possibility is increased local synthesis of E2, which upregulates the ERα receptor.

Our microarray analysis also identifies reduced adipose tissue inflammation in HF pregnant mice. In particular, we observed a pregnancy related decrease in the production of visceral fat *Ccl2* mRNA and in the numbers of adipose tissue pro-inflammatory macrophages in HF-fed but not control diet-fed pregnant animals. This attenuation in inflammation in HF animals may also be driven by increased estrogen action on adipocytes leading to reduced macrophage accumulation into adipose tissue (possibly via reduced chemokine release), given that ovariectomized mice show increased adipose tissue *Ccl2* and *Tnfα* mRNA production which is reversed by estradiol administration [29]. As in our mouse model, a pregnancy-induced attenuation in circulating inflammatory markers has also been shown in a longitudinal study of 240 obese pregnant women [30], although data from our own group shows that an excess of circulating and placentally derived pro-inflamatory cytokines is still present in obese women at term [31]. In parallel with the decrease in adipose tissue mRNA expression of *Ccl2* and *Tnfα* in our HF mice as pregnancy advanced, we also observed a decreased presence of pro-inflammatory adipose tissue macrophages.

We found retinol metabolism, in particular *Rbp4*, to be decreased by pregnancy in HF-fed mice. *Rpb4* is secreted by adipocytes and induces insulin resistance by reducing PI3K signaling in muscle and increasing gluconeogenesis in liver [32]. Thus, in HF-fed mice, reduced *Rbp4* levels in pregnancy could contribute to the alleviation of worsened glucose homeostasis observed in HF-fed compared to control diet-fed mice by E18.5. It is also known that ERα signaling increases Rpb4 expression in 3T3-L1 adipocytes, supporting the central role for estrogen in our phenotype [33]. Leptin levels were increased by pregnancy, despite the decrease visceral adipose tissue and unchanged subcutaneous fat weight, suggesting an alternative source of production, possibly the placenta [18]. Higher leptin levels per unit fat mass may also reflect increased production by adipocytes with increased ERα that are also exposed to a higher concentration of estradiol in pregnancy [34], [35].

In summary, we hypothesize that increased ERα expression contributes to a novel pregnancy-related and fat depot-specific reduction in visceral adiposity and inflammation that counteracts the progressive metabolic perturbations associated with pregnancy. Given the positive association between visceral fat mass and exaggerated cardiovascular risk, the elucidation of the molecular pathway by which ERα suppresses visceral fat accumulation – and the interplay of ERα with the hormonal milieu of pregnancy, might provide insight into therapeutic strategies against metabolic disease [36]. These results provide further support for the rationale for interventions early in pregnancy (or prior to pregnancy) to improve pregnancy outcome.
